# An adjustable gravitational valve for initial VP-shunt treatment in hydrocephalic preterm neonates and infants below 1 year of age

**DOI:** 10.1007/s00381-021-05250-4

**Published:** 2021-06-21

**Authors:** Hans Christoph Bock, Gottberg von Philipp, Hans Christoph Ludwig

**Affiliations:** 1 Department of Neurosurgery, Division of Pediatric Neurosurgery, University Medical Center Göttingen, Robert-Koch-Str. 40, 37075 Gottingen, Germany; 2 Department of diagnostic and interventional Neuroradiology, University Medical Center Göttingen, Robert-Koch-Str. 40, 37075 Gottingen, Germany

**Keywords:** Hydrocephalus, Prematurity, Pediatric, Shunt, Gravitation-assisted valve

## Abstract

**Objective:**

Shunt treatment for hydrocephalus in children should aim for sustainable flexibility in regard to optional, perspective pressure level adjustment during advancing physical and mental development. Gravitation-assisted shunt valves are designed to prevent hydrostatic over-drainage frequently observed in the long course of shunt-treated hydrocephalus. We prospectively studied and analyzed the implication, safety, and feasibility for an adjustable gravitational unit combined with a fixed differential-pressure (DP) valve for neonates and infants primary shunted within the first 12 months of life.

**Methods:**

Clinical course of hydrocephalic neonates and infants who received initial VP-shunt insertion in the early post-natal phase were monitored prospectively on the basis of our digital institutional Hydrocephalus & Shunt Registry. All patients were equipped with a fixed DP valve combined with a programmable gravitational unit activated in upright body position. Patients with a minimum shunt follow-up of 24 months were considered for further statistical analysis regarding hydrocephalus etiology, surgical setting, pre- and post-operative ventricular enlargement, head circumference, valve pressure setting, implication for the adjustment option of the gravitational unit, type and number of shunt complications, and revision-free shunt and valve survival.

**Results:**

Seventy-eight pediatric patients received primary VP-shunt insertion at a mean age of 10 weeks with age gestationally corrected for preterm neonates. Hydrocephalus was related to perinatal IVH (64%), CNS malformation (11%), spina bifida (9%), congenital aqueductal stenosis (9%), and idiopathic (4%) or post-infectious etiology (3%). Fifty-two patients (70%) presented with history of prematurity (gestational age 23–36 weeks). Regular follow-up carried out for a mean period of 63 months demonstrated that ventricular enlargement decreased significantly after applied treatment and excessive head growth could be counteracted effectively. At least one pressure level adjustment was performed in 31% of all patients after 12 months, in 42% after 24 months, and in 64% at the time of last clinical follow-up since initial shunt insertion. Pressure level adjustments were successful in cases of clinical or radiographic signs of under- or over-drainage for individual patients of various ages during entire clinical course. Mean pressure setting for upright position was 24.1 cm H_2_O at the time of initial shunt insertion and increased to 26.4 cmH_2_O at the time of last clinical follow-up. Revision-free shunt-survival rates after 12 and 24 months were 79% and 70% and valve-survival rates 91% and 90%, respectively.

**Conclusion:**

The combination of a fixed DP valve with an adjustable gravitational unit utilized as first-line shunt regimen was feasible and safe in a highly vulnerable subgroup of hydrocephalic infants. The adjustment option for the gravitational unit showed frequent and increasing implication over time and was beneficial even during the very early developmental stage of limited autonomous mobility. To our knowledge this is the first ever reported long-term investigation of an age-consistent pediatric patient collective primary shunted with an adjustable gravitational valve system.

## Introduction

Hydrocephalus (HC) is the main burden of diseases in childhood and shunt procedures are worldwide the most frequent operative therapies [1]. Still and ongoing today shunt revisions are more frequent than the implantation itself, although recent investigations indicate slight improvements regarding shunt malfunctions over the last years [2–4]. The most challenging mission in pediatric neurosurgery and pediatric surgery is the necessary consideration of treatment plans consistent for whole life long. In preterm infancy and perinatal IVH the more or minor destruction of the germinal matrix with blood intake into the ventricles and lesions of the ependymal cell layer is the main cause for lifelong shunt dependency, because the ventricular self-adjustment potential and the subventricular zone harboring stem cells are often terminally disturbed. Surgical and neurofunctional outcome for pediatric hydrocephalus remain issues of substantial importance [5–14] particularly in regard to recent advances in sole or additive neuroendoscopic treatment options.

Implantation of a shunt in the early post-natal phase assumes the shunt system to function patently and correspond to physical parameter alterations of drainage and hydrostatic circumstances during the complete growth period until juvenile and adolescent development. Recent experiences focus on the imminent sustention of the brain’s buoyancy beginning intrauterine and conducted throughout infancy, adolescence, and whole life under all circumstances, body position, awake and during sleep, well-being healthy, and in case of disease [15]. Over-drainage due to shunt treatment is a well-described complication [16–20] and served as an important basis for recent major technical advances in valve design. Regular growth with the alterations of ventricular diameters and progressing skull growth express these alterations which can be seriously disturbed in case of inadequate hydrostatic over-drainage [21]. It is critical for every HC therapy whether performed by neuroendoscopy or with shunt procedures to predict and choose the individual pressure settings of the patient with his disease and history to avoid unphysiological under- or over-drainage [22, 23]. With the aim to prevent upcoming alterations and to be able to provide counter measures, gravity-assisted shunts have been introduced [22], which allow valve resistance adaptations under respect of the horizontal and vertical part of the drainage parameters. This concept reflects the hydrostatic effect of the CSF-filled distal shunt components which tend to withdraw ventricular CSF despite missing existence of a differential pressure gradient. The next evolutionary engineering developmental step was not just to include fixed pressure vertical shunt assistance but to provide a targeted programming of these parameters by developing an adjustable gravitational shunt assistant (proSA®, Miethke). These valves have been introduced from 2009 on into the armamentarium of some of the numerous different valve systems. Within the scope of the PROSAIKA study [24] and other reported case series the adjustable gravitational device proSA® outlined its beneficial effect predominantly for revision surgery in consequence of over-drainage in shunt treated hydrocephalus of various etiology also including young patients and infants [25, 26]. Originally focused on small children to overcome the obstacles of fast growth-related alterations of the valve parameters, meanwhile these valves are more and more used in difficult HC cases, in NPH, and idiopathic hypertension to have an instrument available for easy percutaneous adjustments of the pressure setting.

Since 2012 our institutional first-line treatment setting for shunt-dependent pediatric hydrocephalus patients comprises a fixed differential pressure valve (miniNAV®, Miethke) combined with a programmable, gravitational shunt assistant (proSA® Miethke) which allows adjustments from 0 to 40 cm H_2_O. This selected two-component valve system configuration has currently been replaced by an advanced version (M.blue®, Miethke) combining the fixed differential pressure valve and the adjustable shunt assistant in one single housing. The aim of this study was to follow the idea of a first-line treatment approach for preterm, neonates, and small children up to juvenile and grown-up development. To our knowledge this single-center experience is worldwide unique and allows a considerable overview on this treatment regime. Other centers have already used and reported about gravity-assisted shunt valves in childhood using fixed pressure valves [27–29] or adjustable differential pressure valves alone or in combination with fixed pressure gravitational shunt assistants [30–36]. The aim of our approach was to avoid any shunt revision at a later state, if the stormy body growth develops at the beginning of the second decade by using the regular differential shunt valve in adjunction with an adjustable gravitational unit already at the beginning presuming one valve fits all circumstances. The results of ongoing prospective studies on the safety and efficacy of adjustable gravitational valves are still pending. With the aid of our institutional Hydrocephalus & Shunt Registry [8] we have therefore analyzed prospectively collected data on clinical details, operative procedures, and surgical outcome in a cohort of very young, shunted children equipped with a state-of-the-art differential pressure valve added by adjustable gravity assistance for the first-line treatment attempt.

## Patients and methods

On the basis of our institutional pediatric Hydrocephalus & Shunt Registry [8] we prospectively monitored complete clinical courses of preterm or mature newborns and infants shunted below 12 months of age suffering from hydrocephalus of various etiology between 2009 and 2018. Initial decision for permanent CSF diversion by VPS was based on repeated cranial ultrasound examinations or MRI scans in regard to ventricular enlargement, increasing head circumference, bulging fontanel, or other clinical signs of raised intracranial pressure. All applied shunt systems were equipped with a fixed DP valve (miniNAV®; Miethke, Potsdam Germany) and an adjustable gravitational device (programmable shunt assistant, proSA®). Only patients who presented with a follow-up period of at least 24 months after initial shunt insertion were included for further statistical investigation. Data were taken into account until 31 December 2020 and analysis related to surgical setting, shunt complications, shunt and valve survival, initial pre-operative and latest post-operative ventricular enlargement (fronto-occipital horn ratio, FOHR) and Evans ratio (ER), head circumference, and the utilization of the adjustment-option for the gravitational device 12 and 24 months after shunt insertion and at the time of last clinical follow-up. Measurements of the ventricular enlargement according to FOHR and ER assessment were reviewed blinded and validated with a congruence of >95% by the Department of Neuroradiology University Medical Center Göttingen.

### Shunt surgery

According to our institutional standard procedure, shunt insertion was carried out in endotracheal anesthesia, supine positioning, sterile draping, and perioperative prophylactic antibiosis. Initial individual GAV pressure was validated or adjusted inside the sterile packing with the corresponding adjustment tool directly prior surgical implantation. Ventricular catheters were inserted without navigational guidance predominantly into the right frontal horn of the lateral ventricle over a pre-coronary borehole with the catheter-diverter fixed over the borehole using non-absorbable periostal sutures. Ventricular catheters were connected to a side reservoir (pediatric flushing reservoir) and the downstream DP- and GA-valve positioned subgaleal parietal. Diverting abdominal catheter were inserted ipsilateral paraumbilical using a perforating peritoneal trocar after minimal skin incision and subcutaneous tunneling. In cases of preceding abdominal surgeries with expectable peritoneal scarring a small laparotomy was used or peritoneal catheter insertion was assisted by a pediatric abdominal surgeon. For cranial and abdominal wound closure subcutaneous sutures and epidermal adhesive dressing were used. Only in case of additional contralateral VAD removal (remaining from initial temporizing measures), skin sutures were used here to prevent CSF leakage.

### Post-operative care

Post-operative X-rays (shunt series) were performed to confirm correct implant positioning, adjustment, and integrity of the implant. Post-operative neuroimaging was performed by ultrasonography as long as applicable and CMRI or CCT in case of suspected shunt malfunction, neurological decline, or for follow-up control during further clinical course. All patients received routine follow-up examination 3, 6, 12, and 24 months after initial shunt insertion and additional unscheduled follow-up in case of shunt-related complications. Clinical examinations were performed with the objective to detect any signs of shunt malfunction or implication for re-adjustment of the programmable gravitational device, which was performed accordingly if necessary. All shunt-treated patients underwent subsequent routine follow-ups at least once a year even in case of an unremarkable clinical course. All relevant treatment-related data were managed and immediately documented in our prospective institutional pediatric Hydrocephalus & Shunt Registry application.

### Data analysis

Shunt follow-up (SFU) was defined as the time interval between the date of initial shunt insertion and the date of last clinical follow-up, revision-free shunt, and valve survival (RFSS and RFVS) defined the period between initial shunt insertion and initial shunt or valve revision surgery. Descriptive statistical analysis and Kaplan-Meier survival analysis was carried out by SPSS® Statistic (IBM) and GraphPad Prism Software (San Diego, California). Signed parental informed consent prior to the operative procedure was obtained for all patients and the retrospective data analysis was approved by the ethical review committee of the University Medical Center Göttingen.

## Results

### Patient basic data

**Fig. 1:**
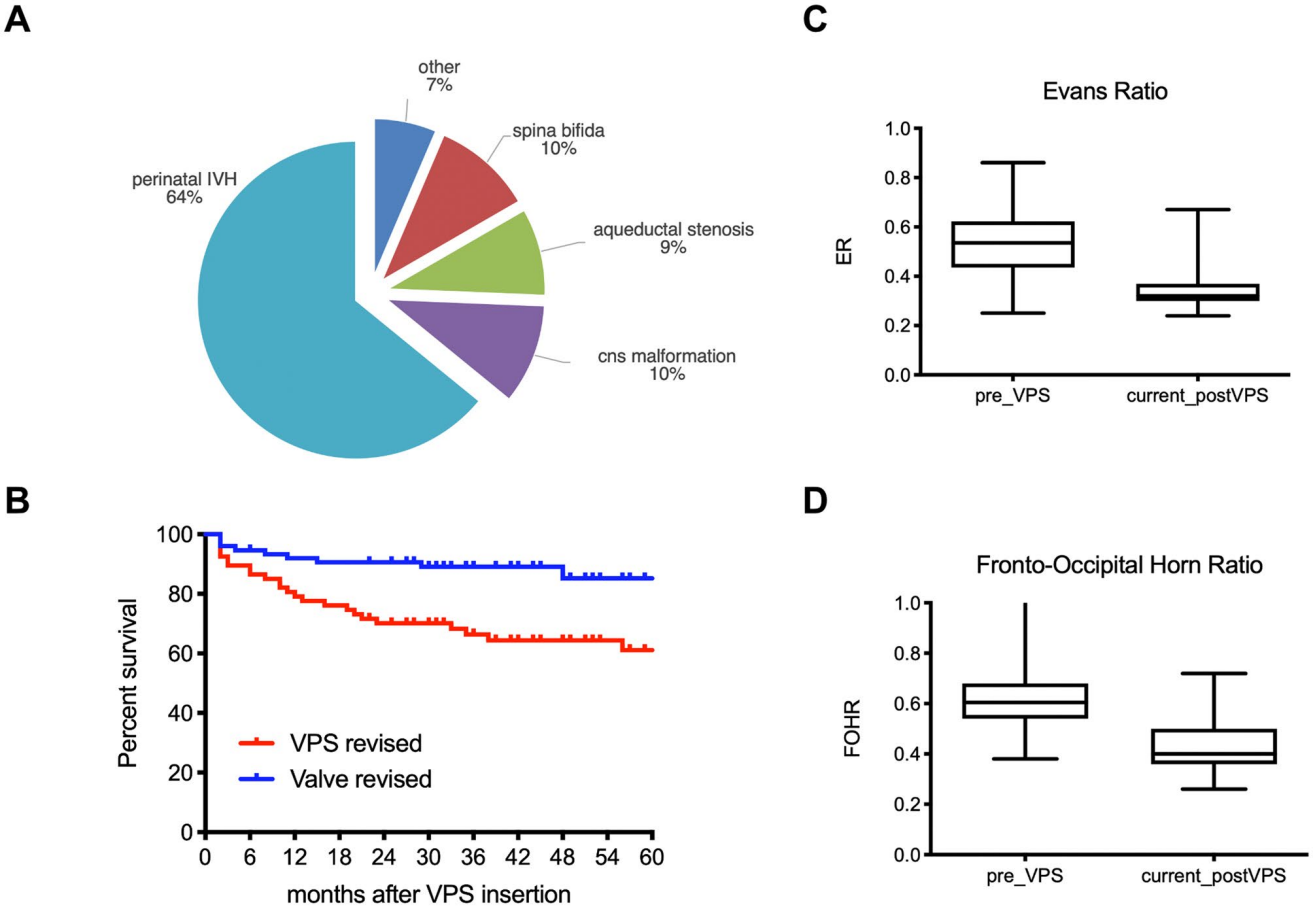
**A** Etiology of hydrocephalus for 74 infants who received early VPS treatment with a fix differential pressure valve and an adjustable gravitational unit. **B** Kaplan-Meier analysis referring to a mean follow-up of 63 months after initial VPS insertion showing revision-free shunt survival rates (79% after 12 months, 70% after 24 months) and valve-survival rates (91% after 12 months and 90% after 24 months) for the entire patient collective. **C** Distribution of Evans ratio and **D** FOHR before, and latest follow-up data after shunt treatment

The patient collective (Fig. 1A) consisted of 78 pediatric patients (61% male, 39% female) with hydrocephalus related to perinatal IVH (64%), spina bifida (10%), congenital aqueductal stenosis (9%), other congenital CNS malformation (10%), or other /idiopathic etiology (7%). Mean age (corrected for preterm neonates) at initial VPS insertion was 10 ±12.8 weeks (range −1.7 to 49 weeks); 15 preterm neonates underwent VPS insertion before completion of 37 gestational weeks. For 50 patients with IVH-related post-hemorrhagic hydrocephalus IVH was found grade 2 in 10 patients (20%) and grade 3 or 4 (PVHI) in 40 patients (80%). Initial diagnostic cranial ultrasound or MRI prior VPS insertion revealed hydrocephalus with FOHR ranging from 0.38 to 0.82 (mean 0.61 ± 0.09) and an ER ranging from 0.25 to 0.86 (mean 0.53 ± 0.13) and was graded mild (19%), moderate (44%), severe (28%), or extreme (9%) prior to shunt treatment (Fig. 1C, D). Latest head circumference before shunt treatment on the basis of age-related percentile showed decompensating growth above the 97th percentile in 28 patients (36%). Increasing head growth but still within the percentile range in 12 (15%), percentile-conform and parallel measurements in 25 (32%), and reduced growth below the third age-related percentile in 13 (17%) of all patients. Overall, 56 patients (72%) presented with a history of prematurity ranging from 23–36 weeks of gestation (mean 29.5 ± 4.2) and a birthweight of 550–3770 g (mean 1579 ± 884 g). Forty-three percent of all patients underwent initial temporizing measures with repeated CSF aspiration via ventricular access device (VAD) prior to shunt surgery due to low body weight and/or inappropriate clinical condition for permanent CSF diversion. Neuro-endoscopic procedures like septostomy [6], ETV [6], cyst fenestration [6], and aqueductal stenting [5] were performed in 7 patients (9%) short-dated prior or in combination with VPS surgery (Table 1).

**Table 1:** Basic patient data

Patient basic data					
		N	%	Range	Mean (±SD)
All patients		78	*100*		
Male		48	*61*		
Female		30	*39*		
Term (≥37 weeks)		22	*28*		
Preterm (<37 weeks)		56	*72*		
Gestation (completed weeks)				23–36	29.5 (±4.2)
Birthweight (g)				550–3770	1579 (±884)
HC etiology					
Perinatal IVH		50	*64*		
CNS malformation		8	*10*		
Spina bifida		8	*10*		
Aqueductal stenosis		7	*9*		
Other		5	*7*		
Initial ventricular dimensions (pre-VPS)					
FOHR				0.38–0.82	0.61 (±0.09)
ER				0.25–0.86	0.53 (±0.13)
Head circumference (pre-VPS)					
Within age-related percentile range		37	*47*		
> 97th percentile		28	*36*		
< 3rd percentile		13	*17*		
VAD temporization prior VPS		28	*36*		
Neuro-endoscopic procedure prior VPS		9	*11*		
Age (corrected for preterm) at initial VPS (weeks)				(−1.7)–49.2	10.1 (±12.7)
Follow-up since initial VPS insertion (months)				24–131	63 (±25.6)
Age at last clinical follow-up (years)				2–11	5.1 (±2.1)

*HC* hydrocephalus, *IVH* intraventricular hemorrhage, *VPS* ventriculo-peritoneal shunt, *FOHR* fronto-occipital horn ratio, *ER* Evans ratio, *VAD* ventricular access device

### Shunt insertion surgery

All shunt insertion surgeries were performed by consultant neurosurgeons with a mean operation time of 47.4 (± 16) min. Peritoneal catheter placement was carried out via minimal skin incision and a peritoneal trocar in 65 cases (88%), via a macroscopic mini-laparotomy and visual control in 4 cases (5%), and via laparotomy with planned assistance of pediatric abdominal surgeons in 5 cases (7%). Antimicrobial impregnated catheters were used in 8 (11%) and perioperative antibiosis (first-generation cephalosporin) in all cases. No intraoperative complications during the surgical procedures were observed. For 77 patients (99%) a 5-cmH_2_O differential pressure valve (DPV, miniNAV®) was connected to a gravitational unit with individually selected pressure setting. The mean initial pressure for the differential pressure valve was 5.0 ± 0.73 cm H_2_O (range 1 to 10) and 19.0 ± 2.9 cm H_2_O (range 10 to 30) for the adjustable gravitational unit (GAV, proSA®). Consequential mean initial pressure setting for upright body position (DPV + GAV) was 24.1 ± 2.9 (range 15 to 35) cm H_2_O (Table 2).

**Table 2:** Data regarding shunt surgery and long-term follow-up

VPS surgery and follow-up data				
	N	(%)	Range	Mean (±SD)
Operation time for initial VPS (min)			17–100	47.4 (±16)
Initial pressure setting (cm H_2_O) horizontal				
Horizontal position (DPV)			1–10	5.0 (±0.73)
Upright position (DPV+GAV)			15–35	24.2 (±2.9)
Pressure setting (cm H_2_O) after 12 months				
Upright position (DPV+GAV)			10–55	24.1 (±5.9)
Pressure setting (cm H_2_O) after 24 months				
Upright position (DPV+GAV)			10–55	24.6 (±6.2)
Latest follow-up pressure setting (cm H_2_O)				
Upright position (DPV+GAV)			9–70	26.4 (±6.2)
Patients with pressure level adjustment				
After 12 months	24	*31*		
After 24 months	33	*42*		
During entire follow-up	50	*64*		
Shunt infection ≤ 30 days after shunt insertion	4	*5*		
Shunt infection anytime within entire follow-up	7	*9*		
Revision-free shunt survival (RFSS) in months			0–131	36.5 (±31.4)
Patients with shunt revision				
After 12 months	25	*32*		
After 24 months	30	*38*		
During entire follow-up	35	*45*		
Revision-free valve survival (RFVS) in months			0–131	55.2 (±30.5)
Patients with valve revision				
After 12 months	9	*12*		
After 24 months	9	*12*		
During entire follow-up	13	*17*		
Latest ventricular dimensions (post-VPS)				
FOHR			0.26–0.72	0.43 (±0.09)
ER			0.24–0.67	0.34 (±0.78)
Head circumference (post-VPS) at last FU				
Within percentile range	51	*65*		
> 97th percentile	7	*9*		
> 3rd percentile	20	*26*		

*DPV* differential pressure valve, *GAV* gravitation-assisted valve, *VPS* ventriculo-peritoneal shunt, *FOHR* fronto-occipital horn ratio, *ER* Evans ratio

### Adjustments of the GAV unit (proSA®)

**Fig. 2:**
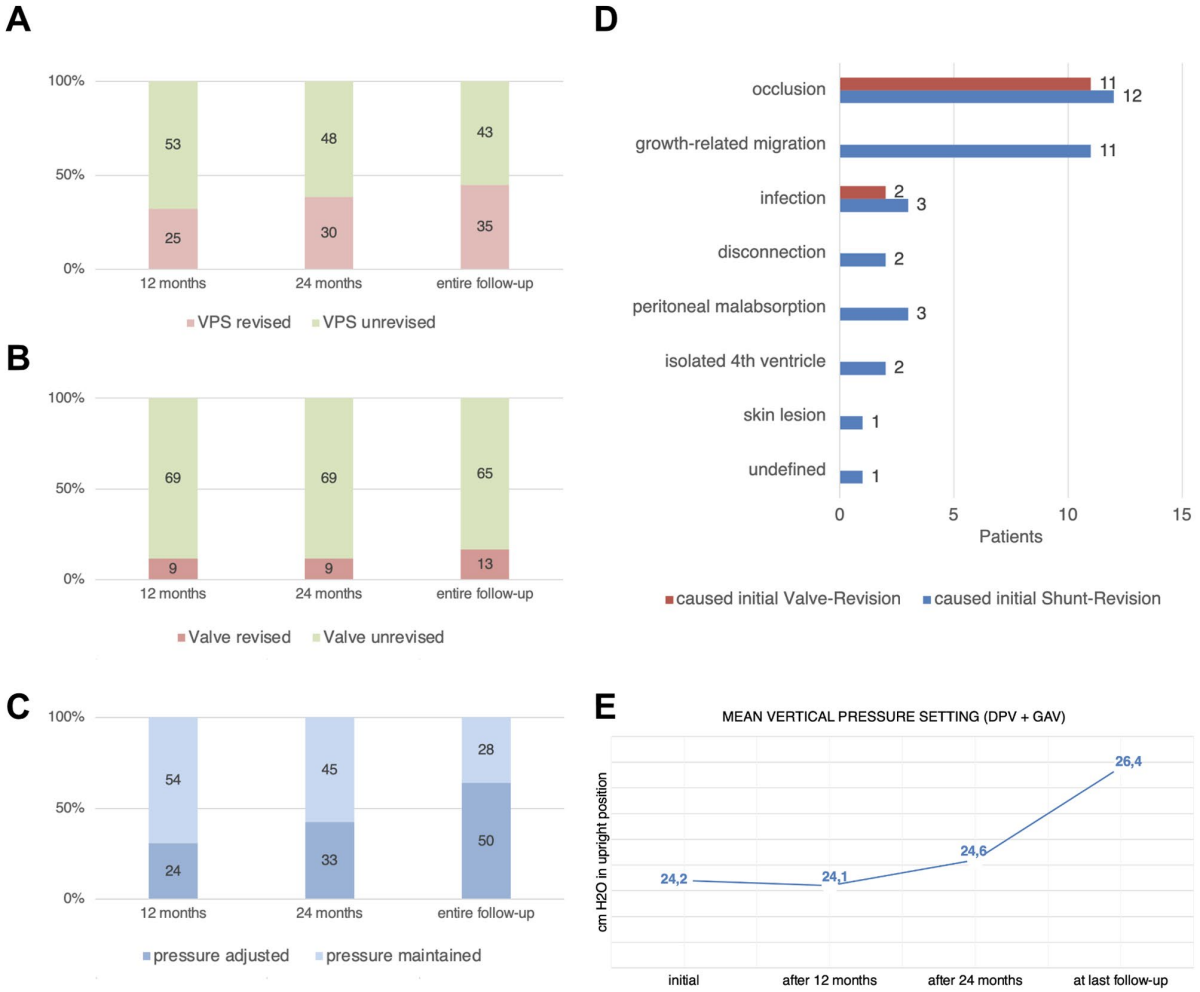
Percentage and number of performed **A** shunt revisions, **B** valve revisions, and **C** GAV-unit pressure level adaptions 12 and 24 months after initial VPS insertion as well as during the entire follow-up period (mean 63 months). **D** Reasons leading to initial shunt- or valve-revision surgery. **E** Alteration of mean pressure setting over time for the sum of DPV and GAV unit corresponding to upright body position

**Fig. 3:**
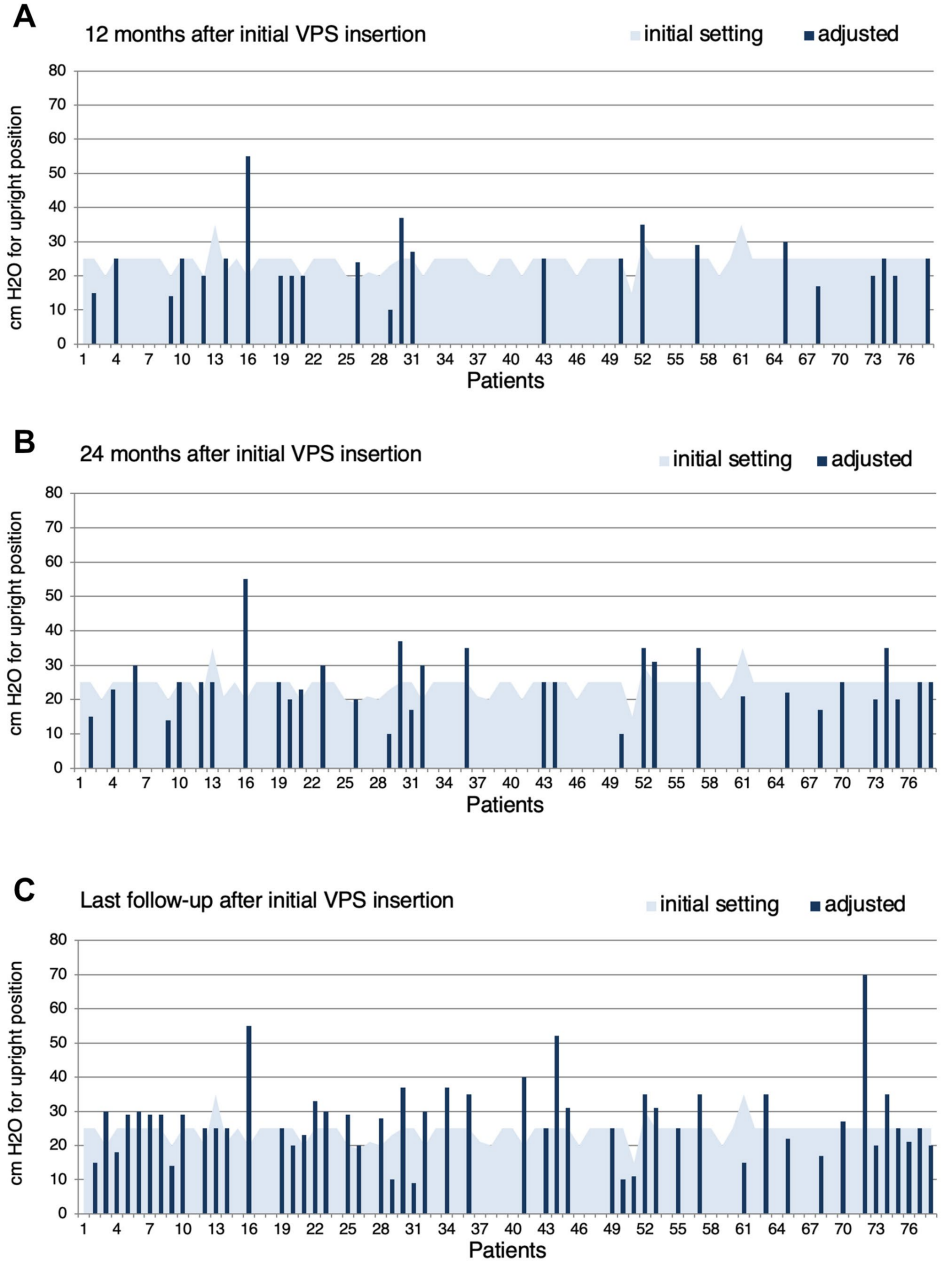
All 78 patient’s initial individual pressure level setting for upright body position (sum of differential pressure + gravitational pressure) illustrated by the light blue skyline and patients with at least one pressure level adjustment after 12 months (**A**), 24 months (**B**), and at the time of last clinical follow up (dark blue columns) (**C**)

Initial pressure setting for the gravitational valve was adjusted by downregulation or upregulation to counteract signs of under- or over-drainage for a confined percentage of patients percutaneous with the corresponding programming tool. No pressure level adjustments as a matter of routine were performed. In total 166 GAV adjustments were carried out during the entire observation period (Fig. 3A–C) and the number of adjustments for individual patients ranged from 0 to 14 (mean 2.1 ± 2.8). Stepwise (2–10 cmH_2_O) GAV adjustment with consequentially altered pressure setting for upright body position after initial shunt insertion (Fig. 2C) had been carried out in 24 patients (31%) after 12 months, in 33 patients (42%) after 24 months, and in 50 patients (64%) at the time of last clinical follow-up (mean 63 months). Comparing the initially selected pressure level and the pressure level at the time of last clinical follow-up, the GAV unit was upregulated in 23 patients (31%), downregulated in 18 (24%), re-adjusted to initial pressure status after varying temporary alteration in 4 (6%), or remained completely unchanged in 29 (39%) of the patient collective. For 12 patients (16%) with signs of under-drainage GAV downregulation was ineffective and revealed underlying VPS malfunction during subsequently performed shunt revision surgery. Mean pressure setting for upright body position (sum of DPV and GAV) changed from initially 24.2 to 24.1 after 12 months, 24.6 after 24 months, and to 26.4 cm H_2_O at the time of last clinical follow-up (Fig. 2E). Quantity of pressure level adjustments for patients with IVH-related hydrocephalus (mean 2.3 adjustments) was increased compared to patients with hydrocephalus of any other etiology (mean 1.2 adjustments).

### Course of ventricular enlargement and head circumference

For 71 patients (96%) a comparative up-to-date neuroimaging examination (10% US, 10% CCT, and 80% MRI) could be provided. For these patients mean time interval between initial pre-VPS and latest post-VPS measurement of the ventricular enlargement ranged between 4 and 92 months (mean 37.4 ± 23.8). FOHR in latest neuroimaging examination ranged from 0.26 to 0.72 (mean 0.43 ± 0.9) and ER from 0.25 to 0.70 (mean 0.36 ± 0.97) showing a significant decrease compared to pre-VPS measures (Fig. 1C, D) and was graded normal (40%), mild (42%), moderate (11%), or severe (3%) after shunt treatment. For 3 patients (4%) no comparative radiographic examination was available at the time of last clinical follow-up. The mean time interval between initial shunt insertion and latest measurement of age-corresponding head circumference was 62 months, ranging from 5 to 131 months. For 28 patients with initially decompensating head circumference above the 97th percentile, head growth at last follow-up was normalized in 20 (71%), stabilized percentile-parallel above 97th percentile in 7 (25%), and showed microcephalic development below 3rd percentile in 1 (4%) patient. For 12 patients with increasing head growth still below or up to the 97th percentile, last follow-up measurements showed stabilized percentile-parallel values within range in 10 (83%) and decreasing development below the 3rd percentile in 2 (17%) patients. For 25 patients with initially percentile-conform measurements, head growth remained unremarkable within percentile range in 21 (84%) and developed microcephalic below the 3rd percentile in 4 (16%). All 13 patients with pre-operative microcephaly below 3rd percentile showed continued percentile-parallel or further decreasing head circumference.

### Shunt complications and revision surgeries

All patients obtained continuous post-operative clinical follow-up for 24–131 months (mean 63 ± 25.6 months). Twelve months after initial shunt insertion 25 patients (32%) and after 24 months 30 patients (38%) underwent shunt revision surgery (Fig. 2A). During entire follow-up 35 patients (45%) underwent at least one shunt revision surgery (range 0–9 revisions, mean 1.3 ± 2.0). For the 35 revised shunts in total (Fig. 2D) the initial shunt malfunction was related to catheter occlusion (34%), growth-related migration (31%), infection (9%), peritoneal malabsorption (8%), disconnection (6%), development of loculated hydrocephalus or isolated fourth ventricle (6%), skin lesion (3%), and undefined cause (3%). Kaplan-Meier analysis revealed revision-free shunt survival rates of 79% after 12 months and 70% after 24 months (Fig. 1B). Surgical shunt revisions involved the valve in 9 patients (12%) after 12 as well as after 24 months and in 13 patients (18%) at last follow-up after VPS insertion (Fig. 2B). In Kaplan-Meier analysis the revision-free valve survival rates were 91% after 12 months and 90% after 24 months (Fig. 1B). Within the subgroup of revised valves, reasons for initial valve revision were dysfunction due to valve occlusion in 11 patients (85%) and shunt infection in 2 patients (15%) (Fig. 2D). Referring to the entire patient collective 3 patients (4%) experienced early post-operative shunt infection within 30 days after initial shunt insertion, total cumulative shunt infections at any time during the entire observation period occurred in 7 patients (9.5%), and accounted exclusively for patients with IVH-related PHHC.

Mean number of shunt revisions during follow-up for the subgroup of IVH-related PHHC (mean 1.6 revisions) was twice as high as for other hydrocephalus etiologies (mean 0.8 revisions). At the time of last clinical follow-up (mean age 5.1 years) 97% of all patients were still or re-equipped with the initially selected type of valve system and a peritoneal CSF diversion; one patient had received an additional fixed pressure shunt assistant and in one patient the fixed DPV was replaced by a programmable DPV (proGAV®). In 2 patients peritoneal CSF diversion failed due to malabsorption and was replaced accordingly by a ventriculo-pleural and a ventriculo-atrial diversion.

## Discussion

The aim of the study was to evaluate safety and feasibility for the primary use of an adjustable gravitational device combined with a fixed differential pressure valve in an extremely vulnerable subgroup of very young pediatric hydrocephalus patients. Young age and a history of prematurity, especially a high incidence of IVH-related PHHC, anticipate a comparatively higher risk of early or repeated shunt failures [5, 6, 31, 37, 38] due to mechanical complications by delicate thin galeal structures, difficult skin closure, and potential risk of catheter migration by concurrent body growth. Of great importance are valve obstructions with extracellular matrix proteins which are highly expressed following denudated ventricular walls with astrocytic scar proliferations [39] In accordance with these circumstances valve materials and shunt management options suitable for this challenging environment and the avoidance of over-drainage during this highly vulnerable period is mandatory. Therefore, questioning the implication and justification for a technically advanced shunt hardware applied for this precarious patient subgroup appears reasonable at first sight. The majority of reports regarding the use of adjustable gravitational valves so far refer to additive implications in shunt treatment to counteract already present over-drainage, involve mainly adult patient collectives, sometimes including children but rarely focus on neonates or infants [24–26].

For pediatric patients it should be of highest priority to aim for a far-sighted and sustainable treatment strategy offering maximal flexibility for optional pressure level adjustments in regard to hydrostatic over-drainage protection. Optimal shunt treatment is focused on a near physiological condition for CSF drainage in regard to the patient’s age, physical constitution, and activity level [22]. Physiological body growth during neonatal, infantile, and juvenile development inevitable leads to various changes especially in hydrostatic parameters and requires the necessity to provide an option for non-invasive adjustment of corresponding shunt-drainage conditions. Considering the vulnerable characteristic of the presented patient collective, our results with revision-free shunt-survival rates of 79% after 12 months and nearly 70% after 24 months enhance the feasibility of this primary treatment protocol even in newborns and infants. Revision-free valve survival for the primary inserted miniNAV and proSA® combination with 90% unrevised valves after 24 months indicate a reliable application in the long term. Shunt malfunction with necessity for revision surgery referred to the well-known and characteristic problems of this patient subgroup regarding vulnerability for infection, growth-related catheter migration, and occlusive dysfunction presumably caused by blood degradation or glial scarring. The rate of performed adjustments (63%) at the time of last clinical follow-up implicates that different subsequent pressure-level alterations in order to adjust CSF drainage for symptom control, sufficient reduction of ventricular enlargement, and stabilization of head growth were conducted to follow an individually adapted treatment protocol. The option to adjust the gravitational unit appears beneficial in our experience even for young infants with limited active vertical mobility. The adjustment option proved increasing implication over the long-term clinical course and physical development from a mainly horizontal towards a predominantly vertical body position. The used valve and shunt technology is only one part of the overall applied therapeutic procedure and early removal of blood constituents together with early and strongly physiological adapted temporizing measures to avoid ependymal loss and glial scarring with extracellular matrix membranes are advocated.

## Conclusion

The utilization of a differential pressure valve in combination with an adjustable gravitational unit in a housing predominantly designed for adults proved good feasibility and safety even for early first-line shunt treatment in preterm neonates and infants in this long-term investigation. The quantitative usage of the GAV-adjustment option increased stepwise parallel to the patient’s advancing physical development and was beneficial for the majority to provide a customized shunt-treatment regimen and simultaneously overcome the risk of over-drainage. Supplementation and combination of neurodevelopmental and quality of life outcome data to detailed surgical outcome parameters in modern pediatric shunt valve treatment appear to be of major importance for conclusive future investigations.
